# ROBBING PETER TO PAY PAUL: HANDLING FINANCIAL CHALLENGES AMONG LOW-INCOME OLDER ADULTS

**DOI:** 10.1093/geroni/igz038.017

**Published:** 2019-11-08

**Authors:** Laura Samuel, Rebecca Wright, Melissa Spahr, Laken C Roberts, Sarah L Szanton

**Affiliations:** 1 Johns Hopkins University School of Nursing, Baltimore, Maryland, United States; 2 Johns Hopkins School of Nursing, Baltimore, Maryland, United States

## Abstract

Low income older adults often face financial challenges which increase their risk for earlier disability and mortality. This study explored the social norms, beliefs and practices relevant to handling financial challenges among low-income community-dwelling older adults residing near Baltimore, MD whom we recruited using convenience and snowball sampling. Four vignette-based focus group sessions included 28 participants. Using hierarchical thematic analysis, three key themes emerged. First, the theme “Rob Peter to pay Paul” describes the consensus that individuals must prioritize financial needs, which required individuals to “work with a budget”, apply for aid, “cry for [aid]” and, when needed, “work something out” with landlords and lenders. One participant described the amount of work by saying “We’re retired but we’re working for ourselves.” Secondly, the theme “Your rent should be first” describes how low income older adults prioritize housing over food and other needs because “resources for housing is a problem” and because homelessness is both more permanent and socially stigmatizing than hunger - “Don’t nobody know you’re hungry unless you tell them, but everybody know when you outdoors.” Finally, the theme “We need to put the word out” describes the consensus that public benefits and community resources should be made more visible and accessible. Many individuals only know about resources because they seek information (“you go and you find out”), but “ it’s hard to ask for help. ” These results can inform the development and improvement of financial and community programs and policies for low-income older adults addressing financial challenges.

